# Cases of Neuralgia, &c.

**Published:** 1823-04

**Authors:** Benjamin Hutchinson


ART. II.—*Cases of Neuralgia, &c.* Communicated by BENJAMIN HUTCHINSON, Esq.

*To the Editors of the London Medical and Physical Journal.*

GENTLEMEN,

I THANK you for the attention you have given to my former communications, and I shall take the liberty of transmitting to you, from time to time, the result of the experience of some of the most eminent and respectable of our brethren, in the treatment of the varied modifications of Neuralgia. I am fully aware that the importance of the subject, and the difficulties opposed to our best-directed remedial efforts in this obstinate class of diseases, present an imperative claim of communication from those practitioners who have fortunately witnessed a successful mode of combating so terrific a foe. Under these impressions, and concurring in opinion with the ingenious editor of the Medico-Chirurgical Review, who, in his candid and liberal criticism of my Essay on Neuralgia, in his quarterly Number for December, says, "We do think that it is incumbent on Mr. Hutchinson to give these cases, or an abstract of them, to the public, through some medium or other;" I shall therefore take the liberty of giving to the profession, through the auspicious medium of your excellently-managed Journal,† the information with which I may be favoured by my friends; and I hope you will allow me, through the same channel, to request the favour of my brethren to furnish me with the results of their practice, should their well-directed means have succeeded by the modes of treatment which I have publicly recommended, or by others, the suggestions of their own experience.

---

† In compliance with the especial request of our valued Correspondent, we have given his communication *verbatim:* otherwise, we are in the habit of omitting all expressions complimentary of ourselves.—EDITORS.

I shall now proceed to transcribe two or three cases of neuralgia, occurring in the practice of eminent men; with the first of which I have been lately favoured by Mr. KILLER, an excellent surgeon in Manchester, and until lately one of the surgeons of that well-regulated institution, the public Infirmary of that populous and wealthy town. I shall take the liberty of copying the communication in the words of Mr. Killer, and of his ingenious partner, Mr. Whatton.

With every sentiment of respect, believe me to remain, Gentlemen, your obedient servant,

BENJ. HUTCHINSON.

*Southwell; February* 1823.

*To B. Hutchinson, Esq.*

"My dear Sir,—In my last I mentioned a case of a painful affection of the face, very strongly resembling tic douloureux. Mr. Whatton, my partner, had visited the lady several times previous to that communication; and, as I was particularly engaged, he took the charge of my patient afterwards: the following, therefore, is Mr. Whatton's statement of the case, and, as I occasionally saw the lady, I can vouch for its minute accuracy. Whatever name be given to the disease, it is very manifest that the remedy which you have so happily and successfully recommended in complaints of this very painful and obstinate character, was highly beneficial; and, if you think it will add any strength to the testimony already presented to the public, you are at liberty to use it as you think proper. With every kind wish, believe me, my dear Sir, yours very faithfully,

"R. W. KILLER."

"*Manchester; December* 2, 1822."

"On the 6th of October, 1822, I was desired to visit Mrs. M. a lady of delicate health, and somewhat irritable constitution, about forty years of age. She had previously been very much exhausted and worn down by a close and affectionate attention upon a near relation lately deceased, who had suffered much through a painful and protracted illness. Mrs. M. complained of a severe and excruciating pain extending over the right side of her face, which prevented her from attending to her usual avocations, and confined her entirely to her chamber. Her pulse were rather below the usual number in the minute, and there was no apparent increase of heat upon the skin, or alteration in the appearance of the tongue. On a supposition that this painful affection might originate in cold, or be occasioned by a carious tooth, I examined her mouth; but nothing was discovered there to account for the disorder: the teeth were all sound, and bore tapping with the finger-nail pretty forcibly, without occasioning any further disturbance or increase of pain. Leeches were applied, and a strong poppy fomentation was directed for present relief, followed by a blister behind the ear. The lady took, with effect, some purgative medicine. On the 7th, she had experienced no relief, and the pain continued as violent as before. The eye and the upper part of the face were swollen, from the irritation of the blister, though without any material additional uneasiness. I saw her again on the following day: no favourable alteration having resulted from the treatment employed, I proceeded to examine the parts more carefully. I found the pain to commence regularly about the centre of the right half of the under jaw, and nearly opposite the anterior maxillary foramen: it continued its course upwards along the cheek, spreading to the right and left, affecting the parts in front of the ear, and extending across to the edge of the nose; and afterwards, proceeding along the fore edge of the temple, spent itself upon the parts surrounding the eye. I found that the paroxysms commenced, with an unvarying regularity, at about half-past nine or ten in the morning, and continued without intermission until three or four in the afternoon; at which time they gradually subsided, and the patient became easier, and at length free from pain. Conceiving this to be a case well adapted for a trial of the subcarbonate of iron, I resolved to lose no time in submitting it to the test. On the 9th, my patient began taking that medicine in doses of a scruple three times a-day. On the second day after using the iron, the paroxysm was greatly diminished both in severity and continuance; and, on the third day, did not commence until one o'clock, gradually subsiding at the usual time. On the fourth, my patient had no recurrence at all, but passed the day comfortably, and without pain. From this time Mrs. M. continued exempt from any regular attack, though she occasionally felt some slight shooting pains along the course of the facial nerves; which, however, were of so short a duration as to excite no uneasiness for the favourable result of the case. Mrs. M. continued the use of the iron steadily until the 20th; at which time, having felt no return of pain for several days, its use was discontinued. This lady has remained entirely free from the disorder, and at the time that I write this is in perfect health.

"The immediate effects of the iron in this lady's case, were a trifling increase of heat and flushing of the face, with some slight acceleration of general vascular action. My patient spoke also of a sensation of drowsiness for some short time after its exhibition. The nerves principally affected appear to have been the infra maxillary, some twigs of the palatine branch of the superior maxillary going to the nose, and the infra orbitar."

With the following very valuable reports I have been favoured by Dr. H. Carter, physician to the Kent and Canterbury Hospital, a practitioner of great ingenuity and very accurate observation, and whose attention has been for some time past directed, in a laudable manner, to the investigation and treatment of the various modifications of neuralgia. In a short letter which lately I had the pleasure of addressing to Dr. Carter, I requested him to favour me with the results of the post-mortem examination of the late lamented Dr. Pemberton, who unfortunately died when under the able and judicious management of Dr. C., and whose dreadful sufferings from long-continued neuralgia faciei are too generally known to the medical faculty to require in this place any particular notice.

The beginning of Dr. Carter's reply to my request on this very important subject, will be perused by my medical brethren with sentiments of the most anxious regret that, in a disease involved in so much perplexing obscurity, the knife of the anatomist is capable of affording us so little satisfactory information.

*To B. Hutchinson, Esq.*

"*Canterbury; Dec.* 5, 1822.

"My dear Sir,—Your letter has just reached me: the great importance of the subject demands an early reply, and I shall endeavour to answer it in the best manner I am able. I am truly sorry to say that the examination of the brain of our mutual friend, Dr. Pemberton, has thrown no light whatever upon the disease which so much interests you. He died with every symptom of pressure. We found the vessels of the brain loaded; though not more so, I think, than I have frequently seen in cases where no cerebral derangement was suspected before death. The nerves, as far as they were traced, (and they were carefully traced as far as the symptoms seemed to require,) exhibited no morbid appearances. I only regret that the cavernous sinusses were not examined, because Mr. Chevalier, in a letter to me since the Doctor's death, has expressed an opinion that the seat of the disease might possibly be there. For my own part, I do not expect that dissection will ever throw much light on this most curious disease. I will now proceed to relate to you a case or two of tic douloureux, in which the subcarbonas ferri was of very distinguished use.

"C. F. ætat. twenty-two; April 30, 1822. A painful affection of her face, which came on several months ago, first on the left side, occupying the lower jaw. It became extremely bad about a fortnight before Christmas. About that time she laboured under cynanche tonsillaris, which went on to suppuration. Her face continued in great pain afterwards for between eight and nine weeks, with considerable swelling of the glands of the neck. She used fomentations and poultices, and suppuration ensued. The pain then quitted the situation which it had hitherto occupied, and attacked the right side of the face, recurring in paroxysms, and affecting the face generally on that side. Her pulse rather frequent and feeble; tongue white, such as is usually observed in persons of a strumous temperament; bowels regular; catamenia the same; appetite good, but every attempt at mastication increases the pain; sleep none. The patient fancied that the complaint was caused by one of the bicuspides of the upper jaw being decayed; but the tooth seemed to be very little injured, and the pain affected the lower as well as the upper jaw.

℞. Subcarbonatis Ferri, ℈ij. Sit pulvis ter die sumendus ex quovis vehiculo idoneo.

℞. Extract. Stramonii,

——— Opii, āā gr. ss.

——— Conii,

——— Hyoscyami, āā gr. ij.

——— Humuli, gr. iij.

Pulv. Jacobi, gr. iij. M. fiant pilulæ iij. omni nocte sum.

"*April* 30.—One powder was taken, and at night the pills. No pain on that night.

"*May* 1.—Powders taken, and pills at night: no pain.—2. Pain returned at about three in the morning, but was confined to the first of the bicuspides of the lower jaw: it occurred twice severely, for about a quarter of an hour each time.—3. Pain at about five in the morning.—4th. Pain at one in the morning.—6th. Pain returned, on the night of the 5th, violently and more general, and was very bad during the whole of the day. Ordered to take four powders a-day, and to continue the pills.—8th. The pain left my patient yesterday about eleven o'clock, and she has experienced but little of it since.—10th. Pain lasted about ten minutes. Ordered to take ʒjss. of the iron three times a-day.—13th. No pain since the last report, only some tenderness of the upper teeth.—20th. A little pain on the 16th, owing, she supposes, to exposure to a cold wind. Bowels have been constipated, and she has taken some laxative pills, which have operated well. Ferri subcarbonas bis die, and the anodyne pills if required.—28th. No return of pain.

"She continued almost entirely free from pain when I saw her in October last; and I called at her master's house this day (December 5th), when my patient assured me that, since last May, she has never been obliged to quit her work for an hour. In cold weather, and especially if she expose herself to the night-air, she experiences some indications of pain, but it is never violent, and ceases on her return to the house.

"I have this day discharged from our county hospital a young woman named Gaum, a person of strumous temperament. She was admitted on account of ophthalmia tarsi, which was cured, but she afterwards was affected with violent intermitting pain of the left sid eof her face. I tried bark and other tonic medicines, with very little effect. At length, October 11th, I gave her Ferri Subcarbonas ℈ij. ter die, and the following pills:

℞. Extract. Stramonii,
——— Opii, āā gr. ss.
——— Hyoscyami, gr. ij.
——— Humuli, gr. v.
Pulv. Glyc. q. s. ut fiant pilulæ iij. accedenti dolore sum.

"She continued this plan for a fortnight, and was rather better. Her bowels being now confined, she was directed to take Decoct. Aloes comp. ʒvj. every morning, or every other morning; and which answered very well.

"*November* 15.—Procedat.—21st. Much better. Procedat.

"*December* 6.—No pain whatever for a week, and she ascribes her cure to the iron; for she has for some time omitted the pills. I now discharged her, with some packets of the medicine to last three weeks: she is directed to leave off the iron by degrees, and to return to the hospital, if the disease again make its appearance.

"In a very obstinate case of neuralgia sciatica, occurring in a young man, who had been treated at St. Thomas's Hospital, but without any success, I was much gratified at the decided benefit derived from the ferri subcarbonas. It was taken in doses of a scruple four times a-day for many weeks, and the patient was discharged from our hospital much better. He remained upon the books as an out-patient for some time; and is now, I understand, free from pain and in very good health.

"Believe me to remain, dear Sir, very faithfully yours,

"HARRY WM. CARTER, M.D."

I feel great propriety in mentioning that, in the same communication, Dr. Carter has given me the history of a severe case of neuralgia faciei, occurring in a young man, aged twenty-six, in which, after the ineffectual trial of various well-known remedies for this dreadful disease, he gave calomel and opium in large doses, and with very decided success. He administered seven grains of calomel and one of opium every night for a fortnight: the effects were a slight affection of his gums, and a removal of his pains. Dr. Carter has also seen the sulphate of quinine useful in one case of neuralgia, occurring in an old lady upwards of eighty years old.

With the history of the following case of neuralgia sciatica I have been favoured by Mr. Forster, fellow of the Royal College of Surgeons, and an eminent practitioner at Southwell.

"William Stokes, of the parish of Bilsthorpe, in the county of Nottingham, about sixty years of age, pale and thin, with a sallow complexion, had experienced, for a considerable length of time, a most severe pain in his back, extending down the thigh in the course of the sciatic nerve, and which was occasionally much aggravated by moving the leg, so as to prevent him pursuing his usual occupation as a day-labourer. Considering this a clearly-marked case of sciatic neuralgia, I resolved immediately upon giving him the subcarbonate of iron, which I had known to have proved eminently successful in several examples of the same disease. I began with the dose of one drachm twice a-day, increasing it to four scruples: this quantity he continued for a month, at which period he began to perceive a very material alleviation of the pain; and, by a perseverance in its use a week or ten days longer, he was enabled to resume his usual employment. Severe and sudden changes of the weather will occasionally produce very slight returns of pain, but never of sufficient severity or continuance to confine him to the house. The powerfully remedial influence of the iron over this painful, and generally very obstinate, disease, was in this case conspicuously manifest. "ROBERT THOMAS FORSTER."

